# The watch-and-wait strategy versus surgical resection for rectal cancer patients with a clinical complete response after neoadjuvant chemoradiotherapy

**DOI:** 10.1186/s13014-021-01746-0

**Published:** 2021-01-19

**Authors:** Qiao-xuan Wang, Rong Zhang, Wei-wei Xiao, Shu Zhang, Ming-biao Wei, Yong-heng Li, Hui Chang, Wei-hao Xie, Li-ren Li, Pei-rong Ding, Gong Chen, Zhi-fan Zeng, Wei-hu Wang, Xiang-bo Wan, Yuan-hong Gao

**Affiliations:** 1 Department of Radiation Oncology, State Key Laboratory of Oncology in South China, Sun Yat-Sen University Cancer Center, Collaborative Innovation Center for Cancer Medicine, 651 Dongfeng Road East, Guangzhou, 510060 People’s Republic of China; 2 Department of Endoscopy, Sun Yat-Sen University Cancer Center, State Key Laboratory of Oncology in South China, Collaborative Innovation Center for Cancer Medicine, Guangzhou, People’s Republic of China; 3 Department of Radiation Oncology, The Sixth Affiliated Hospital, Sun Yat-Sen University, Guangdong Provincial Key Laboratory of Colorectal and Pelvic Floor Diseases, Guangzhou, People’s Republic of China; 4 Key Laboratory of Carcinogenesis and Translational Research (Ministry of Education/Beijing), Department of Radiation Oncology, Peking University Cancer Hospital and Institute, Beijing, People’s Republic of China; 5 Department of Colorectal Surgery, Sun Yat-Sen University Cancer Center, State Key Laboratory of Oncology in South China, Collaborative Innovation Center for Cancer Medicine, Guangzhou, People’s Republic of China

## Abstract

**Background:**

The watch-and-wait strategy offers a non-invasive therapeutic alternative for rectal cancer patients who have achieved a clinical complete response (cCR) after chemoradiotherapy. This study aimed to investigate the long-term clinical outcomes of this strategy in comparation to surgical resection.

**Methods:**

Stage II/III rectal adenocarcinoma patients who received neoadjuvant chemoradiotherapy and achieved a cCR were selected from the databases of three centers. cCR was evaluated by findings from digital rectal examination, colonoscopy, and radiographic images. Patients in whom the watch-and-wait strategy was adopted were matched with patients who underwent radical resection through 1:1 propensity score matching analyses. Survival was calculated and compared in the two groups using the Kaplan–Meier method with the log rank test.

**Results:**

A total of 117 patients in whom the watch-and-wait strategy was adopted were matched with 354 patients who underwent radical resection. After matching, there were 94 patients in each group, and no significant differences in term of age, sex, T stage, N stage or tumor location were observed between the two groups. The median follow-up time was 38.2 months. Patients in whom the watch-and-wait strategy was adopted exhibited a higher rate of local recurrences (14.9% vs. 1.1%), but most (85.7%) were salvageable. Three-year non-regrowth local recurrence-free survival was comparable between the two groups (98% vs. 98%, *P* = 0.506), but the watch-and-wait group presented an obvious advantage in terms of sphincter preservation, especially in patients with a tumor located within 3 cm of the anal verge (89.7% vs. 41.2%, *P* < 0.001). Three-year distant metastasis-free survival (88% in the watch-and-wait group vs. 89% in the surgical group, *P* = 0.874), 3-year disease-specific survival (99% vs. 96%, *P* = 0.643) and overall survival (99% vs. 96%, *P* = 0.905) were also comparable between the two groups, although a higher rate (35.7%) of distant metastases was observed in patients who exhibited local regrowth in the watch-and-wait group.

**Conclusion:**

The watch-and-wait strategy was safe, with similar survival outcomes but a superior sphincter preservation rate as compared to surgery in rectal cancer patients achieving a cCR after neoadjuvant chemoradiotherapy, and could be offered as a promising conservative alternative to invasive radical surgery.

## Introduction

The current standard of care for locally advanced rectal cancer is neoadjuvant chemoradiotherapy followed by total mesorectal excision (TME) [1, 2]. However, TME may at times be associated with an increased risk of complications such as anastomotic leaks [3], and long-term consequences to anorectal and sexual dysfunction [4, 5]. For patients with distal rectal cancer, abdominoperineal resection might be necessitated but at the expense of a permanent stoma and impaired quality of life [6, 7].

In the early 2000s, reports have shown that approximately 15–27% [8–10] of patients who underwent neoadjuvant chemoradiotherapy followed by radical surgery could achieve a pathological complete response (pCR), which was associated with favorable long-term outcomes [10]. This stimulated interest to explore the possibility of omitting surgery in such patients. In 2004, Habr-Gama and colleagues pioneered the first report comparing an observational strategy against invasive surgery following chemoradiation in a group of rectal cancer patients who achieved a clinical complete response (cCR) [11]. Since then, discussions on the “watch-and-wait” strategy have been fueled by a series of studies [12–19] which have demonstrated similar survival results but better functional outcome [12] as compared to surgery. Although local recurrence rates were higher in the watch-and-wait group, most of these recurrences were salvageable and local disease control after salvage surgery was satisfying [14].

However, the existing medical literatures concerning the watch-and-wait strategy has been limited by small sample sizes, single-cohort studies [14–16, 18], and studies consisting of comparative analyses focusing mainly on comparing patients with a cCR managed with the watch-and-wait strategy against patients with a pCR identified upon radical surgery [11–13, 19]. In regard to single cohort studies, the risks of the watch-and-wait strategy may have been under-investigated due to a lack of comparative analyses. Furthermore, it has been reported that 61% of patients with a pCR did not have the performance of a cCR [20], thus the group of patients with a pCR are, to some extent, different from the group of patients with a cCR. As patients with a pCR usually have a better prognosis with a low risk of distant metastasis [10], they are not the perfect control group in discussing the choices for patients with a cCR.

Until now, there has been no published data from randomized trials regarding the safety and efficacy of the watch-and-wait strategy, and concern about the risk of distant metastases during this conservative treatment approach remains [19]. A prospective randomized control trial assigning clinical responders to surgery or watch-and-wait to compare their oncologic outcomes would be ideal to shed light on this debate but such randomization is difficult in real-world clinical practice.

In the present study, we investigated the long-term clinical outcomes and the risk of distant metastasis in rectal cancer patients achieving cCR using real-world multicenter data by performing comparative analyses between patients managed under the watch-and-wait strategy and those who underwent surgical resection.

## Method

### Study population

This was a multicenter retrospective cohort study. Data were provided by physicians from three centers in China. A total of 117 sequential patients with stage II/III rectal adenocarcinoma who were identified as achieving a cCR after neoadjuvant chemoradiotherapy and adopted the watch and wait strategy from Sept 1, 2010 to Jan 30, 2018 were included in the study. Patients were staged based on the 7th edition of the American Joint Committee on Cancer Staging Manual [21]. In order to select the comparison group, 354 patients who achieved a cCR after neoadjuvant chemoradiotherapy but underwent standard TME surgery in the same time period were also identified. Patients were excluded if they had: (1) prior transanal excision or (2) coexistence of a second primary malignant disease within a 5-year period. Part of the patient data were published in previous studies [22–24].

**Fig. 1:**
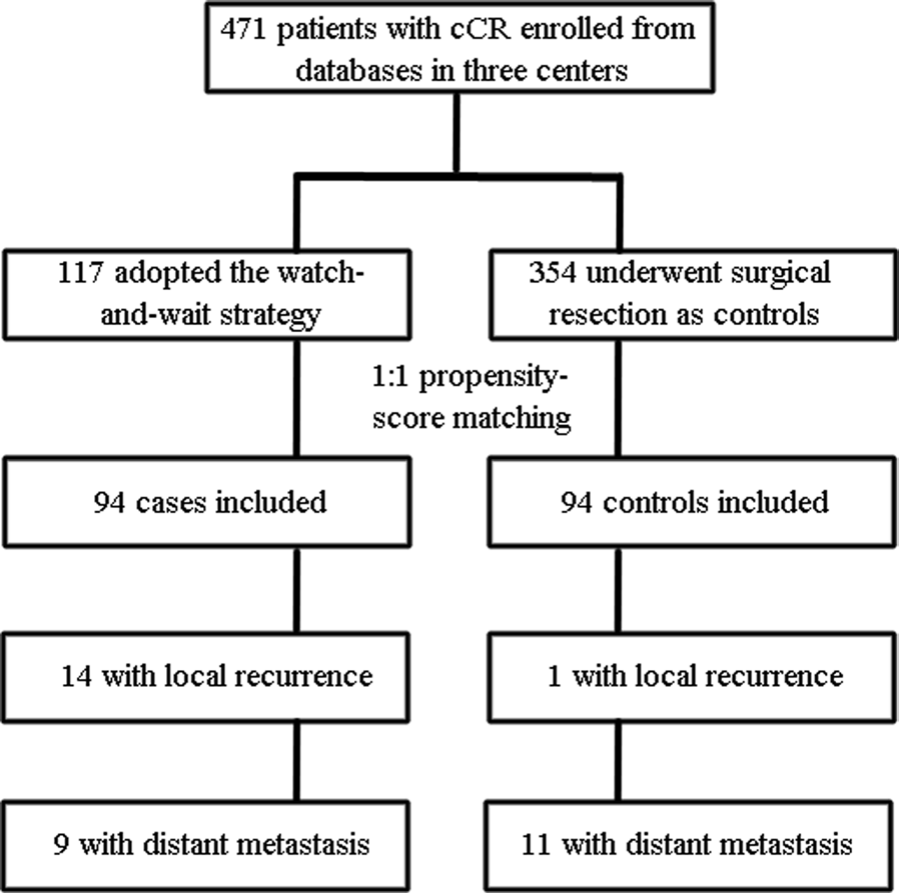
Schematic illustration of the study cohort

To reduce potential confounding effects between the watch-and-wait and surgical group, propensity score matching analyses were performed in a 1:1 nearest neighbor caliper of width 0.1. The propensity score was estimated using logistic regression based on the baseline variables of age, sex, T stage, N stage (N0 or N +), WHO performance status, carcinoembryonic antigen (CEA) level, location of the tumor and chemotherapy regimen used (with or without oxaliplatin). In the matched cohort, 94 patients in the watch-and-wait group and 94 patients in the surgical group were included (Fig. 1).

### Study procedure

Patients’ data were collected from clinical chart review, physician records, patient correspondence, and telephone interviews. Treatment-related parameters mainly included dose of radiotherapy and chemotherapy, evaluation of cCR, and type of surgery. Follow-up assessments were performed every three months in the first two years after treatment and every six months in the following three years. The last date of follow-up was Jan 30, 2019.

### Assessment of cCR

All endoscopic images and pelvic magnetic resonance imaging (MRI)/computed tomography (CT) scans of patients at the time of reassessment from each center were reviewed. Information regarding digital rectal examination, and chest and abdominal CT scans were extracted from medical records. cCR was evaluated by findings including digital rectal examination, colonoscopy, and radiographic images. Diagnosis of a cCR was based on the following criteria: (1) no palpable nodule upon digital rectal examination; (2) no residual tumor or a flat white scar with or without telangiectasias and no ulceration or nodularity in endoscopic findings [25]; (3) no residual tumor and no suspicious lymph nodes on MRI or pelvic CT scans; and (4) absence of distant metastasis.

### Outcome measurement

Our primary endpoint was disease specific survival (DSS), defined as the time from diagnosis until the date of death from rectal cancer. Our secondary endpoints were non-regrowth local recurrence-free survival (LRFS), distant metastasis-free survival (DMFS), non-regrowth disease-free survival (DFS) and overall survival (OS). Local recurrence was defined as recurrence in the pelvis and distant metastasis was defined as recurrence outside the pelvic region. For patients in the watch-and-wait group, local recurrence after diagnosis of a cCR that could be salvaged afterward usually have good prognoses and are distinguishable from local recurrence after TME surgery; this was defined as local regrowth [18]. DFS was defined as survival without local recurrence and distant metastasis, and non-regrowth DFS of the watch-and-wait group were only included events after salvage surgery. OS was defined as survival till the date of death from any cause. All time-to-events were calculated from the date of diagnosis until the time of their occurrence or censored at last follow-up, and death of other diseases not related to cancer were not considered as event for LRFS, DMFS and DFS in the current study.

### Statistical analysis

Categorical variables were described in terms of frequency, and compared using the χ2 test. Non-normally distributed continuous variables were described as medians, and compared using the Mann–Whitney U test. LRFS, DMFS, DFS, DSS and OS were calculated using the Kaplan–Meier method with log rank test. Multivariate analyses were performed using the Cox proportional hazards model. R software (version 3.2.2) was used for propensity score matching, with the package of “MatchIt” and “Optmatch”. Statistical analyses were performed using the Statistical Package for the Social Sciences Program (SPSS, Chicago, IL, version 24.0). Significance was defined as a two-sided *P* < 0.05. This study was retrospectively registered at clinicaltrials.gov.

## Results

### Baseline characteristics

The baseline characteristic of the entire cohort was provided (Additional file 1: S1). After matching, the differences in age, sex, WHO performance status, T stage, N stage, location of the tumor and level of CEA were not significant (Table 1). Sex distribution showed that 106 (56.4%) patients were male in the matched cohort. The median age at diagnosis was 56.5 (interquartile range [IQR], 48.0–63.0) years.

**Table 1:** Characteristics of patients according to treatment group (matched cohort)

	Surgical group (*N* = 94)	Watch-and-wait group (*N* = 94)	*P*
Age-median (IQR), year	56.0 (49.0–62.3)	57.5 (46.0–65.0)	0.760^b^
Sex			
Male	51 (54.3%)	55 (58.5%)	0.556^a^
Female	43 (45.7%)	39 (41.5%)	
WHO performance status-*N *(%)			0.665^a^
0	54 (57.4%)	49 (52.1%)	
1	39 (41.5%)	43 (45.7%)	
2	1 (1.1%)	2 (2.1%)	
T stage-*N* (%)			0.883^a^
T2	8 (8.5%)	9 (9.6%)	
T3	71 (75.5%)	68 (72.3%)	
T4	15 (16.0%)	17 (18.1%)	
N stage-*N* (%)			0.385^a^
N0	24 (25.5%)	19 (20.2%)	
N1/2	70 (74.5%)	75 (79.8%)	
Distance from anal verge-median (IQR), cm	4.0 (3.0–5.0)	4.0 (3.0–5.0)	0.996^b^
CEA-median (IQR), ng/ml	2.86 (1.71–4.77)	2.82 (1.61–4.74)	0.770^b^
Radiation dose, median (IQR), Gy	50 (50–50)	50 (50–50)	0.686^b^
Chemotherapy regimen-*N* (%)			0.875^a^
Capecitabine or fluorouracil only	29 (30.9%)	30 (31.9%)	
Capecitabine or fluorouracil plus oxaliplatin	65 (69.1%)	64 (68.1%)	
Therapeutic patterns in the neoadjuvant setting-*N *(%)			
Concurrent chemoradiotherapy only	21 (22.3%)	26 (27.7%)	0.501^a^
Concurrent chemoradiotherapy plus induction and/or consolidation chemotherapy	73 (77.7%)	68 (72.3%)	

Data are showed in *N* (%) or median (interquartile range [IQR])

^a^*P* values were determined by χ2 test

^b^*P* values were determined by Mann–Whitney U test

### Treatment

Radiotherapy was given in standard fractions, most frequently with a total dose of 50 Gy. All patients received fluorouracil based chemoradiotherapy. A total of 68 (72.3%) patients in the watch-and-wait group received induction and/or consolidation chemotherapy, while 84 (89.4%) patients in the surgical group received perioperative chemotherapy in addition to chemoradiotherapy. All patients received at least one chemotherapeutic drug, capecitabine or 5-flurouracil. Among them, 129 (68.6%) patients were prescribed oxaliplatin in combination with capecitabine or 5-flurouracil.

All patients in the surgical group received TME and had postoperative negative resection margins. In all, 48 (51.1%) patients were diagnosed as having a pCR. The median time to surgery from the end of chemoradiotherapy was 57 days (IQR, 50–66). A total of 32 (34.0%) patients had distal tumors that required an abdominoperineal resection and permanent colostomy.

Endoscopic images at baseline and after chemoradiotherapy were reviewed in all patients. Pelvic MRI was performed in most patients (175/188 at baseline, and 178/188 after chemoradiotherapy). In those patients who didn’t have MRI, pelvic CT was performed instead due to being declared medically unfit or other reasons.

### Survival

**Fig. 2:**
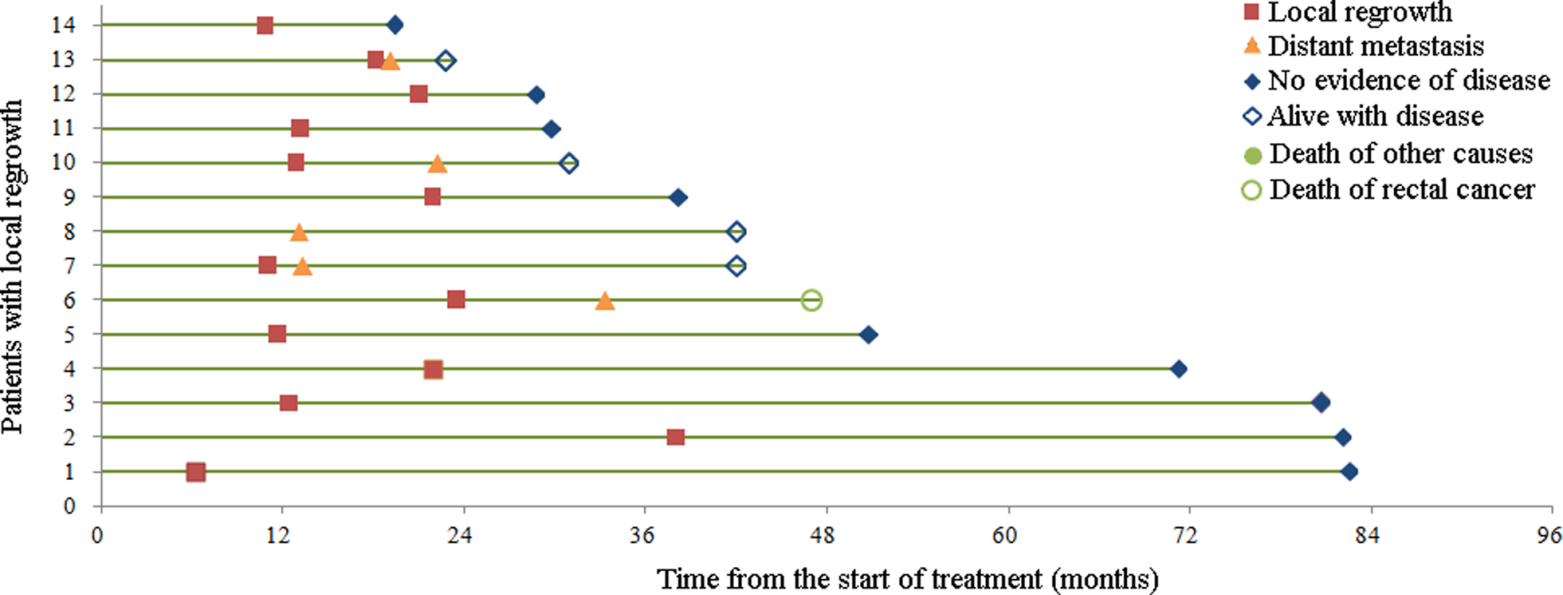
Swimming plot of patients with local regrowth (*N* = 14)

The median follow-up time for the whole cohort was 38.2 months (IQR, 25.9–52.4). During the follow-up period, 14 patients in the watch-and-wait group had local tumor regrowth (all of whom were confirmed through biopsies), 13 (92.9%) were located in the bowel wall and one (7.1%) was located in regional lymph nodes. Most local regrowth occurred in the first two years from the start of treatment, of whom five (35.7%) were in the first year and eight (57.1%) were in the second year (Fig. 2). Of the 14 patients with local regrowth, 12 (85.7%) had salvaged R0 resection, of whom five underwent the Miles procedure. The 3-year non-regrowth LRFS was 98% (95% CI 95–100%). In contrast, one patient in the surgical group had local recurrence, and couldn’t receive salvaged R0 resection. Three-year non-regrowth LRFS was 98% (95% CI 95–100%) for the surgical group, which was not significantly different from the watch-and-wait group (*P* = 0.506). Ultimately, sphincter preservation was achieved in 87 (92.6%) patients in the watch-and-wait group and 62 (66%) in the surgical group, respectively. For patients with a tumor located within 3 cm of the anal verge, the watch-and-wait group had obvious advantage compared with the surgical group in sphincter preservation (89.7% vs. 41.2%, *P* < 0.001).

**Fig. 3:**
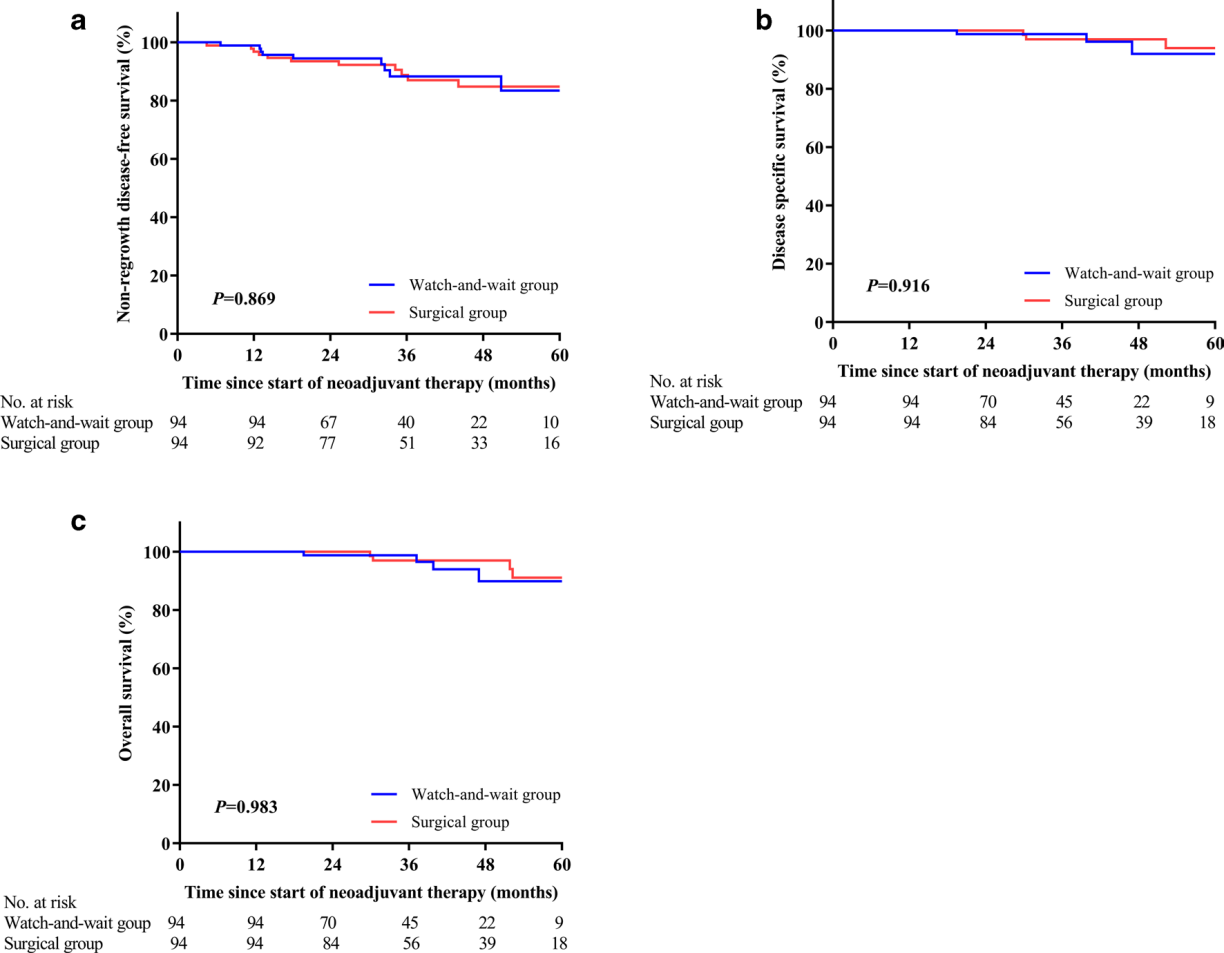
Non-regrowth disease-free survival (**a**), disease specific survival (**b**) and overall survival (**c**) for patients in the matched groups

During the same period, a total of twenty patients in the watch-and-wait and surgical groups developed distant metastases (Additional file 1: S2), respectively. Subgroup analysis indicated that, of the 14 patients with local regrowth in the watch-and-wait group, five developed distant metastases, while only four of the 80 patients who sustained a cCR had distant metastases. Of the five patients with both local regrowth and distant metastases, three had concurrent distant metastases detected within three months before or after the diagnosis of local regrowth, while two patients developed distant metastases followed by local regrowth. In the surgical group, four patients who were diagnosed as having a pCR and seven patients who were diagnosed as non-pCR developed distant metastasis (4/48 vs. 7/46, *P* = 0.35). Three-year DMFS was 88% (95% CI 80–96%) in the watch-and-wait group and 89% (95% CI 82–96%) in the surgical group (*P* = 0.874), while three-year non-regrowth DFS was 88% (95% CI 80–96%) in the watch-and-wait group and 89% (95% CI 82–96%) in the surgical group (*P* = 0.869, Fig. 3a). The Cox regression model was used to test whether the watch-and-wait strategy was associated with more distant metastasis, using T stage, N stage, CEA level, location of the tumor and regime of chemotherapy (oxaliplatin added or not) as covariates, and the difference was non-significant (HR 0.741, 95% CI 0.295–1.866, *P* = 0.525). The median time to development of metastases in the twenty patients was comparable between the watch-and-wait and surgical groups (22.3 months vs 17.9 months, *P* = 1.000).

Ten patients died during the follow-up period (four from the watch-and-wait group), of whom seven were due to cancer progression, with the other three patients dying from diseases unrelated to cancer. The 3-year DSS was 99% (95% CI 96–100%) and 97% (95% CI 93–100%) for the watch-and-wait and surgical groups (*P* = 0.916), respectively, and the corresponding 3-year OS was 99% (95% CI 96–100%) and 97% (95% CI 93–100%) (*P* = 0.983), respectively (Fig. 3b, c).

Analysis in the unmatched cohort also showed no significant difference between the two groups (Additional file 1: S3, Additional file 1: S4). The 3-year DSS was 99% (95% CI 97–100%) and 96% (95% CI 94–98%) for the watch-and-wait and surgical groups (*P* = 0.643), respectively, and corresponding 3-year OS was 99% (95% CI 97–100%) and 96% (95% CI 94–98%) (*P* = 0.905), respectively.

## Discussion

The watch-and-wait strategy applied with close follow-up was shown to be feasible for stage II/III rectal cancer patients achieving a cCR after chemoradiotherapy, without significant a compromise of survival outcomes and resulted in a better sphincter preservation rate compared to that in patients who underwent radical surgery.

The local regrowth rate in this study was consistent with [16, 19] or slightly lower than those of previous reports [15,^ 17^, 18]. This may have been due to the stringent criteria for the selection of patients with a cCR in the present study, in whom pelvic MRI was performed in ~ 94% of the patients (vs. only 71% in a study by Van der Valk and colleagues). Similar to other studies [14, 18], most cases of local regrowth in our study occurred within the first two years, were predominantly located within the bowel wall, and were salvageable. Ultimately, the local recurrence rate after salvage surgery in the watch-and-wait group was comparable to that of the surgical group, which further indicated that delayed surgery may not actually affect the local control of patients achieving a cCR.

It is noteworthy that the proportion of local regrowth in the watch-and-wait group (14/94) was much lower than the non-pCR rate in the surgical group (46/94). This can be explained by the following hypotheses. Although macroscopically diagnosed as a cCR, the surgical cases diagnosed as non-pCR may still have had clinically undetectable residual tumor at the time of reassessment but could have regressed in the succeeding months if the patients had undergone the watch-and-wait protocol, probably turning into a pCR. As demonstrated in previous studies, the value of biopsy in identifying a cCR is still unclear [26, 27] and the ideal timing for biopsy warrant further exploration. Thus, biopsy was not mandated as a criterion for selecting cases with a cCR.

Apart from local recurrences, the main concern for the watch-and-wait strategy was the risk of distant metastasis during follow-up. The distant metastasis rate of the watch-and-wait group in this study (9.6%) was comparable to the results of Habr Gama (8.9%) [14], Van der Valk (8%) [18] and Smith JJ (8%) [19]. In general, the distant metastasis rate in patients achieving a cCR were much lower than those with similar pretreatment tumor stages [28], indicating that tumors achieving a cCR could have less aggressive biological behavior and are associated with favorable prognoses. In addition, there was no significant difference in distant metastasis rates between the watch-and-wait and surgical groups, demonstrating the overall clinical feasibility of the watch-and-wait approach.

However, we did observe a trend for a higher distant metastasis rate in patients with local regrowth as compared to those who sustained a cCR in the watch-and-wait group, which was in agreement with previous reports [19]. Whether the delay of surgery in these patients was the source of metastasis remains to be elucidated. For the watch-and-wait group, patients who had sustained a cCR but still developed distant metastases, it can be deduced that surgical resection could not have possibly prevented the occurrence of these events, indicating undetectable preexisting micrometastases might have been the underlying cause. However, in patients with both local regrowth and distant metastasis, it was difficult to determine whether there was a causal relationship. In this study, we found that the median time to development of distant metastasis and DMFS rate in patients of the watch-and-wait group was similar to that of the surgical group. These findings indicate that distant metastasis may occur before the decision of surgery or watch-and-wait. Furthermore, rather than a delay in surgery, a more aggressive biological tumor profile may be present in the tumor with local regrowth compared to those with a sustained cCR, resulting in this observed type of treatment failure. Thus, a higher distant metastasis rate observed in patients with local regrowth should not be considered to be a hurdle affecting treatment decisions.

### Limitations

To the best of our knowledge, this study comprised of one of the largest cohorts of patients with a cCR and provided a direct comparison between choices for these same patients. However, there were still some limitations worth mentioning. First, this retrospective design may have had inherent selection bias. In the year before 2015, adopting the watch-and-wait strategy were mainly based on personal choice, and this might have resulted in the imbalance between the two treatment groups. However, this did reflect the true situation of real-world clinical practice, and a more balanced comparison was also made in the matched groups. Second, the cCR assessment was performed by different physicians in three centers at different times, and inconsistencies in the confirmation of a cCR might have existed. Third, there were no data concerning the quality of life between the two groups in the current study, so it was hard to deduce whether the higher sphincter preservation rate could translate into better functional outcomes. Finally, the results of a matched comparative analysis should always be interpreted with caution, and more validation cohorts are needed to verify the safety of this type of non-operative management.

## Conclusion

As compared to standard TME, the watch-and-wait strategy was found to be clinically feasible and demonstrated a superior sphincter preservation rate for stage II/III rectal adenocarcinoma patients achieving a cCR after neoadjuvant chemoradiotherapy. However, large prospective cohort studies with functional outcomes are warranted to further confirm these observations.

## Supplementary Information

**Additional file 1.** **S1:** Characteristics of patients according to treatment group (non-matched cohort). **S2:** Number and location of distant metastasis in the matched groups. **S3:** Distant metastasis-free survival (A), non-regrowth disease-free survival (B), disease specific survival (C) and overall survival (D) for patients in the watch-and-wait group (N = 117). **S4:** Local recurrence-free survival (A), distant metastasis-free survival (B), disease-free survival (C), disease specific survival (D) and overall survival (E) for patients in the surgical group (N = 354).

## Data Availability

Research data are stored in an institutional repository and will be shared upon request to the corresponding author.
