# Supplementary material for: The watch-and-wait strategy versus surgical resection for rectal cancer patients with a clinical complete response after neoadjuvant chemoradiotherapy
Source: Radiat Oncol. 2021 Jan 19;16:16. doi: 10.1186/s13014-021-01746-0 (PMC7816381; doi:10.1186/s13014-021-01746-0)

**Online-Only Supplements**

S1. Characteristics of patients according to treatment group (non-matched cohort).

S2: Number and location of distant metastasis in the matched groups.

S3: Distant metastasis-free survival (A), non-regrowth disease-free survival (B), disease specific survival (C) and overall survival (D) for patients in the watch-and-wait group (*N* =117).

S4: Local recurrence-free survival (A), distant metastasis-free survival (B), disease-free survival (C), disease specific survival (D) and overall survival (E) for patients in the surgical group (*N* =354).

**S1: Characteristics of patients according to treatment group (non-matched cohort).**

|  | Surgical group (*N* =354) | Watch-and-wait group (*N* =117) | *P* |
| --- | --- | --- | --- |
| Age, median (range), year | 56 (25-83) | 58 (29-80) | 0.116 ^b^ |
| Sex |  |  |  |
| Male | 224 (63.3%) | 70 (59.8%) | 0.504 ^a^ |
| Female | 130 (36.7%) | 47 (40.2%) |  |
| WHO performance status- *N* (%) |  |  |  |
| 0 | 207 (58.5%) | 61 (52.1%) | 0.334 ^a^ |
| 1 | 137 (38.7%) | 54 (46.2%) |  |
| 2 | 10 (2.8%) | 2 (1.7%) |  |
| T stage- *N* (%) |  |  |  |
| T2 | 16 (4.5%) | 13 (11.1%) | 0.012 ^a^ |
| T3 | 259 (73.2%) | 87 (74.4%) |  |
| T4 | 79 (22.3%) | 17 (14.5%) |  |
| N stage- *N* (%) |  |  |  |
| N0 | 87 (24.6%) | 24 (20.5%) | 0.369 ^a^ |
| N1/2 | 267 (75.4%) | 93 (79.5%) |  |
| Distance from anal verge, median (IQR), cm | 5 (3.3-6.8) | 4 (2.3-5.0) | 0.000 ^b^ |
| CEA- median (IQR), ng/ml | 3.49 (1.84-7.1) | 2.56 (1.55-4.37) | 0.003 ^b^ |
| Radiation dose, median (IQR), Gy | 50 (50-50) | 50 (50-50) | 0.541 ^b^ |
| Chemotherapy regimen- *N* (%) |  |  |  |
| Capecitabine or fluorouracil only | 63 (17.8%) | 48 (41.0%) | 0.000 ^a^ |
| Capecitabine or fluorouracil plus oxaliplatin | 291 (82.2%) | 69 (59.0%) |  |

Data are showed in *N* (%) or median (interquartile range), unless otherwise stated.

^a^ *P* values were determined by χ2 test.

^b^ *P* values were determined by Mann-Whitney U test.

^c^ Number and percentage of patients received radiotherapy with a total dose of 45-50.6Gy.

**S2: Number and location of distant metastasis in the matched groups.**

| **Location of distant metastasis** | **Number of cases** | |
| --- | --- | --- |
|  | **Watch-and-wait group** | **Surgical group** |
| Lung | 7 | 5 |
| Lymph node | 1 | 3 |
| Liver | 3 | 2 |
| Bone | 0 | 1 |
| Brain | 0 | 1 |
| Peritoneum | 0 | 1 |

**S3: Distant metastasis-free survival (A), non-regrowth disease-free survival (B), disease specific-survival (C) and overall survival (D) for patients in the watch-and-wait group (*N* =117).**


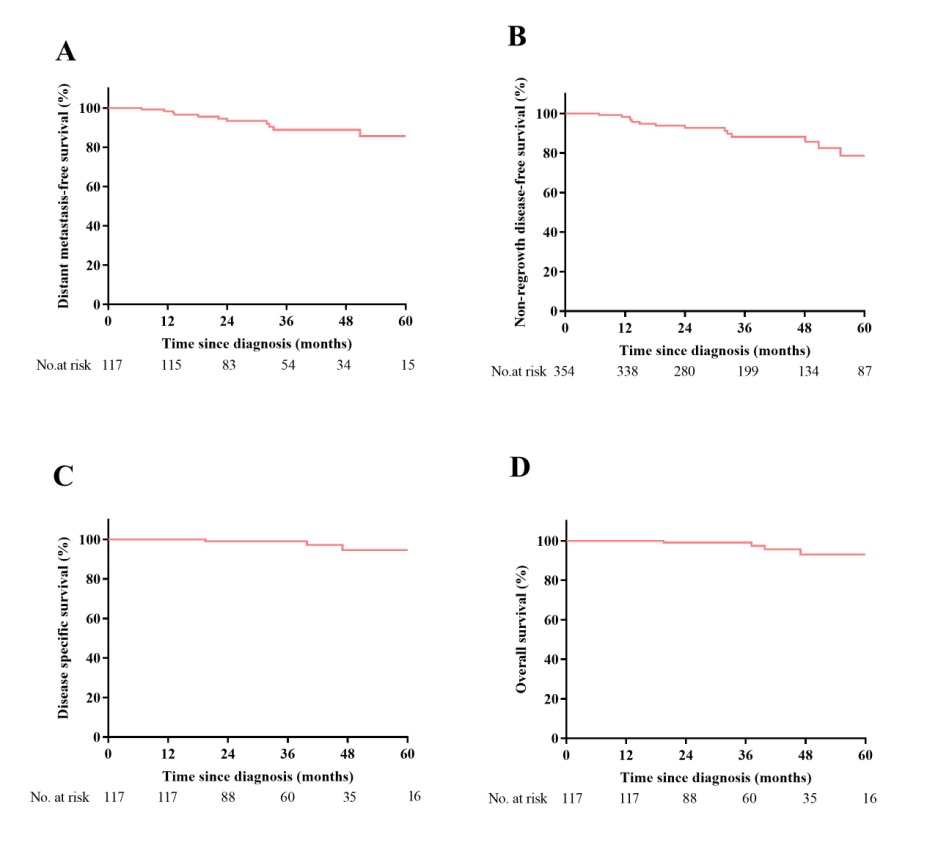


**S4: Local recurrence-free survival (A), distant metastasis-free survival (B), disease-free survival (C), disease specific-survival (D) and overall survival (E) for patients in the surgical group (*N* =354).**


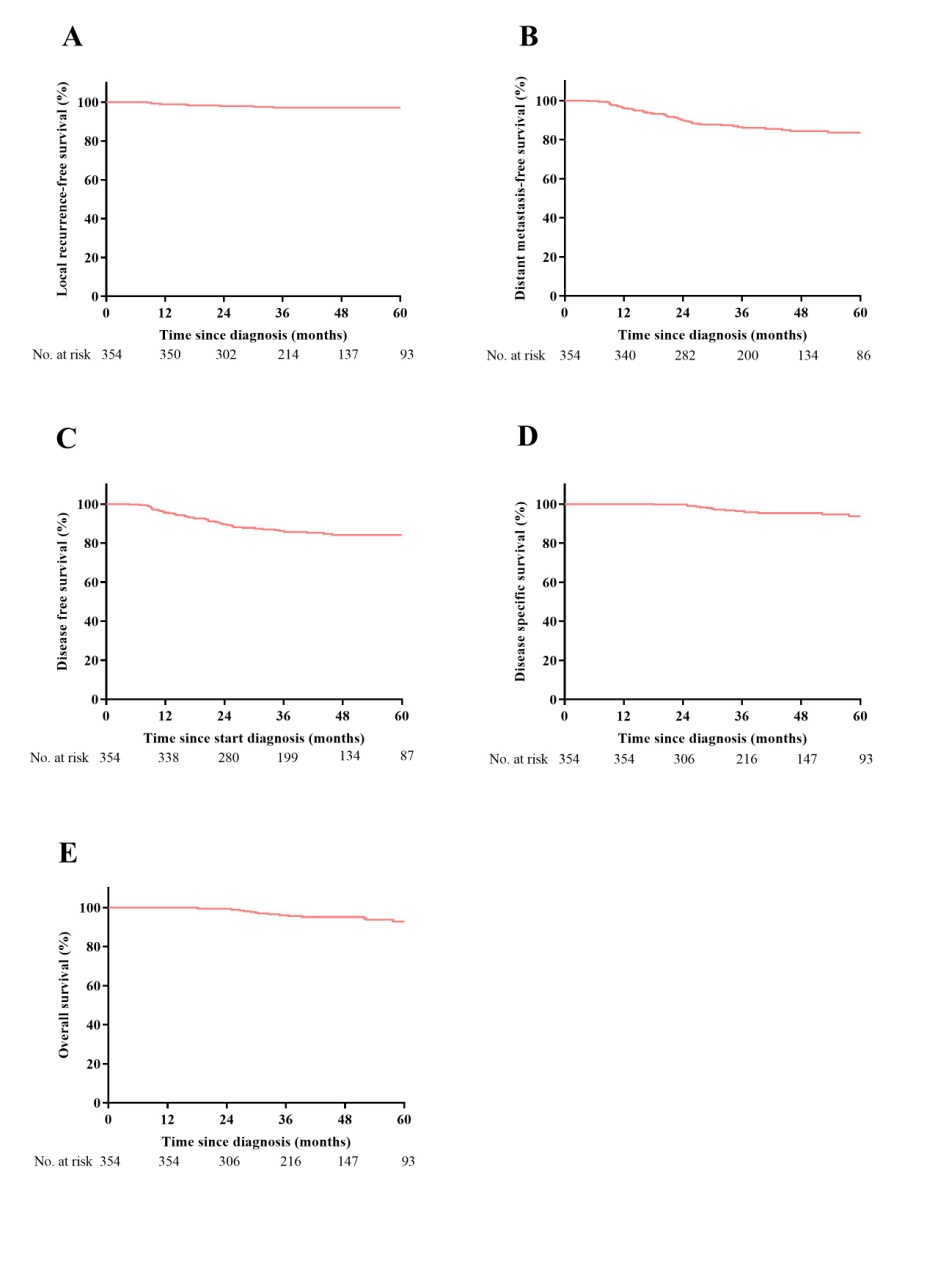

Supplement: Supplementary file 1 — Additional file 1. S1: Characteristics of patients according to treatment group (non-matched cohort). S2: Number and location of distant metastasis in the matched groups. S3: Distant metastasis-free survival (A), non-regrowth disease-free survival (B), disease specific survival (C) and overall survival (D) for patients in the watch-and-wait group (N = 117). S4: Local recurrence-free survival (A), distant metastasis-free survival (B), disease-free survival (C), disease specific survival (D) and overall survival (E) for patients in the surgical group (N = 354). [file 13014_2021_1746_MOESM1_ESM.docx]
